# A Neural Network Model for Intelligent Classification of Distal Radius Fractures Using Statistical Shape Model Extraction Features

**DOI:** 10.1111/os.70034

**Published:** 2025-04-03

**Authors:** Xing‐bo Cai, Ze‐hui Lu, Zhi Peng, Yong‐qing Xu, Jun‐shen Huang, Hao‐tian Luo, Yu Zhao, Zhong‐qi Lou, Zi‐qi Shen, Zhang‐cong Chen, Xiong‐gang Yang, Ying Wu, Sheng Lu

**Affiliations:** ^1^ Department of Orthopedic Surgery The First People's Hospital of Yunnan Province, The Affiliated Hospital of Kunming University of Science and Technology Kunming Yunnan China; ^2^ The Key Laboratory of Digital Orthopaedics of Yunnan Province Kunming Yunnan China; ^3^ Department of Orthopedics 920th Hospital of Joint Logistics Support Force, PLA Kunming China; ^4^ The Faculty of Medicine, Nursing, and Health Sciences, Monash University Australia; ^5^ Key Lab of Statistical Modeling and Data Analysis of Yunnan, Yunnan University Kunming China; ^6^ Department of Orthopaedics Peking Union Medical College Hospital, Peking Union Medical College and Chinese Academy of Medical Sciences Beijing China

**Keywords:** artificial intelligence, computer‐aided diagnosis, distal radius fracture, principal component analysis, statistical shape model

## Abstract

**Objective:**

Distal radius fractures account for 12%–17% of all fractures, with accurate classification being crucial for proper treatment planning. Studies have shown that in emergency settings, the misdiagnosis rate of hand/wrist fractures can reach up to 29%, particularly among non‐specialist physicians due to a high workload and limited experience. While existing AI methods can detect fractures, they typically require large training datasets and are limited to fracture detection without type classification. Therefore, there is an urgent need for an efficient and accurate method that can both detect and classify different types of distal radius fractures. To develop and validate an intelligent classifier for distal radius fractures by combining a statistical shape model (SSM) with a neural network (NN) based on CT imaging data.

**Methods:**

From August 2022 to May 2023, a total of 80 CT scans were collected, including 43 normal radial bones and 37 distal radius fractures (17 Colles', 12 Barton's, and 8 Smith's fractures). We established the distal radius SSM by combining mean values with PCA (Principal Component Analysis) features and proposed six morphological indicators across four groups. The intelligent classifier (SSM + NN) was trained using SSM features as input data and different fracture types as output data. Four‐fold cross‐validations were performed to verify the classifier's robustness. The SSMs for both normal and fractured distal radius were successfully established based on CT data. Analysis of variance revealed significant differences in all six morphological indicators among groups (*p* < 0.001). The intelligent classifier achieved optimal performance when using the first 15 PCA‐extracted features, with a cumulative variance contribution rate exceeding 75%. The classifier demonstrated excellent discrimination capability with a mean area under the curve (AUC) of 0.95 in four‐fold cross‐validation, and achieved an overall classification accuracy of 97.5% in the test set. The optimal prediction threshold range was determined to be 0.2–0.4.

**Results:**

The SSMs for both normal and fractured distal radius were successfully established based on CT data. Analysis of variance revealed significant differences in all six morphological indicators among groups (*p* < 0.001). The intelligent classifier achieved optimal performance when using the first 15 PCA‐extracted features, with a cumulative variance contribution rate exceeding 75%. The classifier demonstrated excellent discrimination capability with a mean AUC of 0.95 in four‐fold cross‐validation and achieved an overall classification accuracy of 97.5% in the test set. The optimal prediction threshold range was determined to be 0.2–0.4.

**Conclusion:**

The CT‐based SSM + NN intelligent classifier demonstrated excellent performance in identifying and classifying different types of distal radius fractures. This novel approach provides an efficient, accurate, and automated tool for clinical fracture diagnosis, which could potentially improve diagnostic efficiency and treatment planning in orthopedic practice.

## Graphical abstract

This study developed an automated distal radius fracture classification system based on statistical shape model (SSM) feature extraction and neural network classification. The method first extracts point cloud data from CT images, then extracts fracture features through registration, downsampling, and PCA dimensionality reduction, before inputting them into a neural network for classification. The system achieved 97.5% classification accuracy for normal bones, Barton fractures, Colles fractures, and Smith fractures in the Chinese population, providing an efficient and accurate auxiliary diagnostic tool for clinical practice.

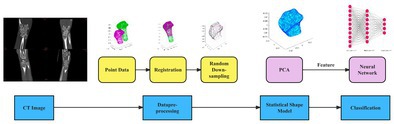

**Abbreviations**
AI: artificial intelligence
AUC: area under the curve
CNN: convolutional neural networks
DICOM: digital imaging and communication in medicine
DRFs: distal radius fractures
NN: neural networks
PCA: principal component analysis
ROC: receiver operating characteristic
SSM: statistical shape model

## 1 Introduction

Statistical shape model (SSM) is a statistical image analysis technique that quantifies morphological changes through principal component analysis (PCA) [1, 2, 3]. Compared to traditional measurements, SSM allows a comprehensive analysis of skeletal shape patterns [2, 3, 4]. SSMs have been successfully applied in various orthopedic applications, including predicting femoral head coverage and assessing fracture risks [4, 5, 6].

Artificial intelligence (AI), particularly neural networks (NNs) and convolutional neural networks (CNNs), has shown promising results in medical image analysis [7, 8, 9]. While CNNs have been widely applied in fracture detection across various anatomical sites [9, 10, 11, 12, 13, 14, 15, 16, 17], they typically require large training datasets and manual feature selection [18, 19, 20, 21].

Distal radius fractures (DRFs), accounting for 12%–17% of all fractures [22, 23], can lead to significant complications if not properly treated [24, 25, 26]. In emergency settings, missed diagnoses of DRFs are particularly common (29% of all misdiagnosed hand/wrist fractures) due to high workloads and inexperience among non‐specialists [27]. These diagnostic challenges highlight the need for automated classification systems to assist clinicians in accurate fracture identification and typing.

While conventional radiographs can assess basic parameters, CT imaging provides superior visualization of rotational deformities and articular surface integrity [23, 24, 25]. Recent studies have shown that CNN‐based models can detect DRFs with high accuracy [22, 26, 28], with ensemble models achieving 97.03% accuracy and 95.70% sensitivity, outperforming both orthopedic surgeons (93.69%) and radiologists (92.53%) [29]. However, these models are limited to fracture detection and cannot classify fracture types [30].

This study proposes a novel approach combining 3D‐SSM with NN for DRF classification. Unlike conventional CNN‐based methods [31, 32], our approach: Automatically extracts morphological features through SSM; compared to conventional CNN methods requiring thousands of images, our SSM‐based approach can achieve effective classification with dozens of samples due to its dimensionality reduction nature (from 2000 points to 15 principal components); Enables both detection and classification of DRFs.

The purpose of this study was to evaluate the feasibility and effectiveness of this combined SSM‐NN approach for intelligent DRF classification, potentially offering a more efficient and accurate diagnostic tool.

**FIGURE 1:**
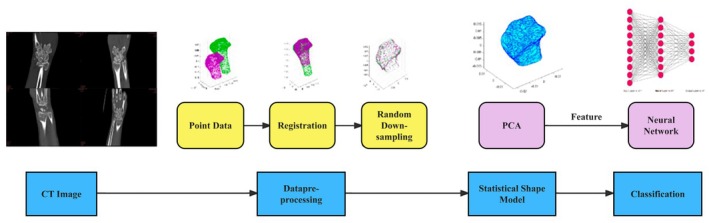
Flowchart of materials and methods.

The purposes of this study were: (i) to develop and validate a novel combined statistical shape model and neural network (SSM‐NN) approach for distal radius fracture classification; (ii) to evaluate the accuracy and reliability of this intelligent classification system in distinguishing different types of distal radius fractures (Colles', Barton's, and Smith's fractures); and (iii) to assess the potential clinical application value of this automated classification tool in improving diagnostic efficiency (Figure 1).

## 2 Methods

### 2.1 Participants and Preprocessing

In this study, we collected computed tomography (CT) digital imaging and communication in medicine (DICOM) data from August 2022 to May 2023, including 43 normal distal radius and 37 distal radius fractures (DRF, including 17 Colles fractures, 12 Barton fractures, and 8 Smith fractures). Inclusion criteria were as follows: (1) healthy radius between 16 and 75 years of age without deformity or dislocation; (2) radiological examination and CT examination by a senior physician; and (3) confirmed diagnosis of Colles, Barton, and Smith fractures. Exclusion criteria were as follows: (1) bone tumor; (2) carpal dislocation; (3) radial deformity; and (4) osteoarthritis.

This study was approved by the Ethics Committee of the 920th Hospital of Joint Logistics Support Force of PLA (approval number: 2019‐018(Other)‐02).

Patient information was retrieved and CT data was downloaded through the image archiving and communication (PACS) system. Forty‐three CT images of healthy wrists were included in the study, with patients ranging in age from 16 to 71 years (mean age 34 ± 14 years), with 34 males and 9 females, all of whom had left‐sided wrists. Also included in this study were CT data of 37 patients with distal radius fractures (DRF) with an age range of 9–81 years (mean age 44 ± 20 years), of whom 18 were males and 19 were females, 17 were right‐sided wrists and 20 were left‐sided wrists. Of these DRF patients, 17 Colles fractures, 12 Barton fractures, and 8 Smith fractures. This dataset was provided by XXX (model: Siemens Somatom Definition 2008G), with the following image sequence parameters: 512 × 512 pixels; reconstruction layer thickness, 0.75 mm; and image format, DICOM 3.0.

Distal radius 3D reconstruction of CT sequences in software Mimics 21.0 (Materialize NV, Leuven, Belgium). The preprocessing of point cloud data followed a systematic approach. After initial 3D reconstruction in Mimics 21.0, we performed standardized alignment using the proximal radius as the reference plane. The initial registration process included fundamental operations such as rotation, mirroring, and translation to achieve preliminary alignment of the distal radius models. To address individual variations and point cloud differences, we implemented a point‐to‐plane iterative closest point (ICP) algorithm with carefully selected parameters. The algorithm was configured with a convergence threshold of 1e‐6, maximum iterations of 100, and a distance rejection threshold of 2.5 mm. This configuration effectively minimized the point‐to‐plane distances between corresponding points in the target and reference clouds. Registration quality was continuously monitored using root mean square error (RMSE), maintaining a threshold of 0.5 mm to ensure accuracy. To optimize computational efficiency while preserving anatomical accuracy, we employed a downsampling scheme using a voxel grid filter with a voxel size of 1 mm. This process resulted in approximately 2000 points per participant's final point cloud, providing an optimal balance between computational efficiency and geometric detail retention. Throughout the process, experienced orthopedic doctors verified four key anatomical landmarks (A: sigmoid notch volar lips; B: sigmoid notch dorsal lips; C: radial styloid process vertex; D: the highest point on the dorsal side of Lister's tubercle) through visual inspection and measurement to ensure anatomical accuracy [30]. Based on these landmarks, six anatomical parameters were measured: four distance measurements (AB, bc, AC, CD in mm) and two angular measurements (ALF: the angle between the lunate facet line and radius long axis; ASR: the angle between sigmoid notch midpoint‐styloid process line and radius long axis). The fracture detection criteria were based on quantitative measurements of these parameters, with specific thresholds established for different fracture types (Colles': dorsal angulation > 20°; Smith's: volar angulation > 20°; Barton's: articular surface displacement > 2 mm). The dimensions of the registered point cloud datasets were inconsistent; therefore, the sub‐sampling scheme obtained the dimensions of all participants' fusions to maintain the image originality without redundant information.

### 2.2 Statistical Shape Model

The construction of our SSM followed a systematic four‐step approach. First, during data preprocessing, we standardized the CT data orientation, performed initial 3D reconstruction, and generated point clouds containing approximately 2000 points per sample. Subsequently, point cloud registration was achieved using the point‐to‐plane ICP algorithm, with a convergence threshold of 1e‐6, a maximum 100 iterations, and a distance rejection threshold of 2.5 mm. In the third step, we established correspondence through automatic landmark identification, followed by nearest neighbor matching and rigorous error checking and validation. Finally, statistical analysis was performed to complete the SSM construction.

**FIGURE 2:**
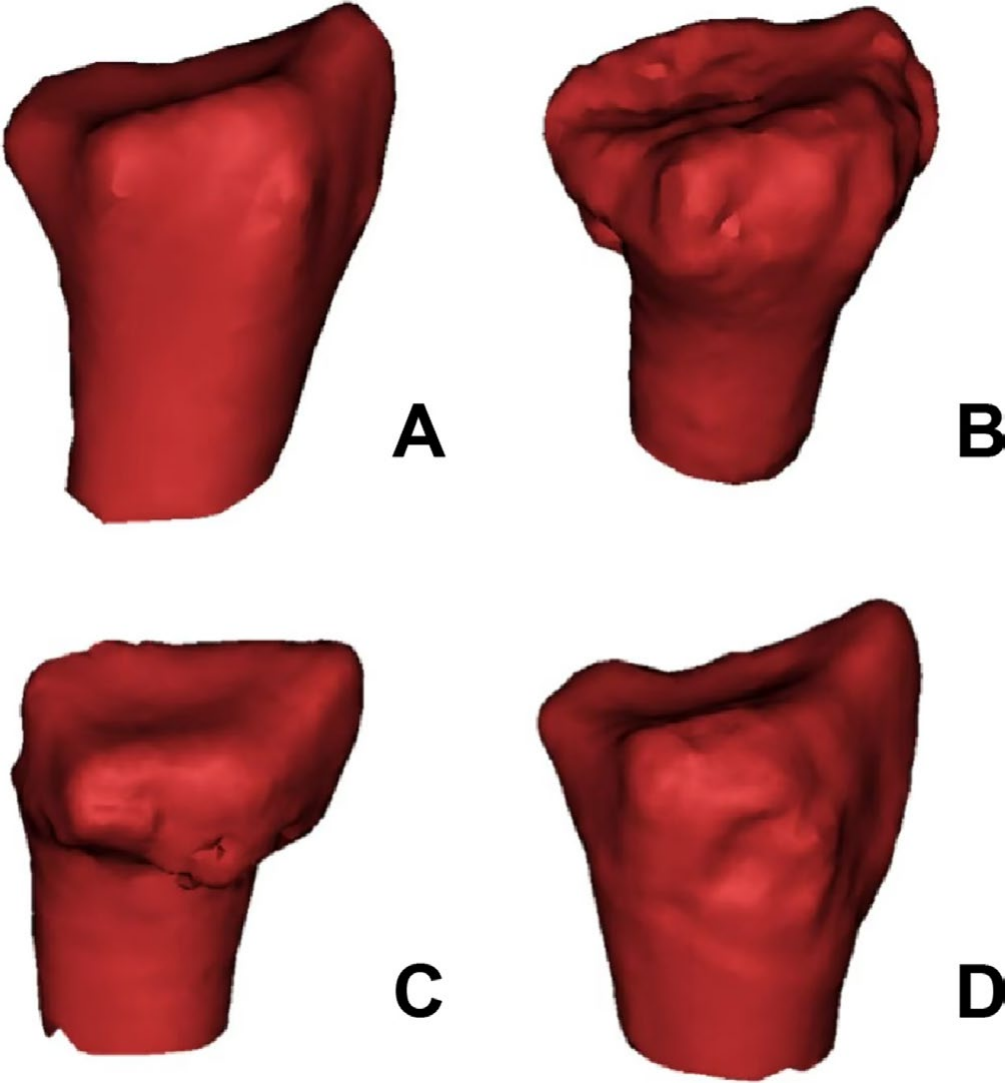
The SSM of different groups (A) Normal; (B) Barton's fracture; (C) Colles' fracture; (D) Smith's fracture.

Here, we describe SSMs that was used to represent objects in images. The shape of an object was represented by a set of M points, which could be of any dimension (2D coordinate *M* × 2 dimensions matrix and 3D coordinate *M* × 3 dimensions matrix). The shape was the quality of the point configuration that was invariant under certain transformations. Generally, when moving, rotating, or scaling an object, the object's shape was invariant. The latest advances in shape statistics allow the statistical technique to create SSMs that can analyze shape differences and changes (Figure 2).

The dataset was point cloud data, which has disordered characteristics. This brought significant challenges to establishing the SSM. Hand annotation for landmarks was a good choice and could be consistently located from one image to another.

Therefore, we automatically consider the nearest Euclidean distance between both points to adjust the order of point clouds.

For instance, in a 3D image, we represented the *M* points $\left\{T_{\mathrm{ij}}:\left(x_{\mathrm{ij}},y_{\mathrm{ij}},z_{\mathrm{ij}}\right),i=1,\ldots ,N,j=1,\ldots ,M\right\}$, where *N* was the number of participants, and *M* was the number of points for each participant. Based on the target *i*th, each point traversed the points of other participants $\left\{\left(x_{-\mathrm{il}},y_{-\mathrm{il}},z_{-\mathrm{il}}\right),-i=1,\ldots ,i-1,i+1,\ldots ,N,l=1,\ldots ,M\right\}$, and selected the point in the nearest location as the corresponding point.

$$d_{-\mathrm{il}}=\sqrt{{\left(x_{-\mathrm{il}}-x_{i.}\right)}^{2}+{\left(y_{-\mathrm{il}}-y_{i.}\right)}^{2}+{\left(z_{-\mathrm{il}}-z_{i.}\right)}^{2}}$$

We found $\min_{j}\;d_{-i.}$, where $d_{-i.}=\left(d_{-i1},\ldots ,d_{-\mathrm{iM}}\right)$, then l=j.

PCA is an effective method commonly used in SSMs. We first reduced the data dimension from *M* × 3 to a level that was easier to calculate and manage. The approach was as follows: we have *N* sets of points $\left\{T_{i}:\left(x_{i.},y_{i.},z_{i.}\right),i=1,\ldots ,N\right\}$, where $T_{i}$ was the vector in the 3*M ×* 1 dimension space. The mean and covariance of data points were defined as follows:

$$\bar{T}=\frac{1}{N}\sum_{i=1}^{N}\;T_{i}\;,S=\frac{1}{N-1}\sum_{i=1}^{N}\;\left(T_{i}-\bar{T}\right){\left(T_{i}-\bar{T}\right)}^{⊤}$$

The mean value of the data represented the data's concentration and reflected the population's overall morphological characteristics. The variance represented the data dispersion and reflected population characteristics and individual differences. PCA used matrix decomposition to solve eigenvalues and eigenvectors. Each principal component was independent and strongly correlated. In SSM, the target matrix chose the covariance matrix, and the eigenvalues λ and eigenvectors ϕ were computed as follows:

$$S\phi_{k}=\lambda_{k}\phi_{k}$$

where $\phi_{k}^{⊤}\phi_{k}=1$, k=1,…,3M and $\lambda_{1}>\lambda_{2}>\ldots >\lambda_{3M}$. A larger $\lambda_{k}$ value corresponded with a more significant $\phi_{k}$ change pattern. We extracted the eigenvectors corresponding to the front t eigenvalues to approximate the variation degree of the participants. The cumulative contribution rate was as follows:

$$\sum_{k=1}^{t}\;\lambda_{k}/\lambda_{T}>\alpha ,\;0\leq \alpha \leq 1$$

where $\lambda_{T}=\sum_{k=1}^{3M}\;\lambda_{k}$, α represented the interpretation of the reduced dimension model and the proportion of the original model. The SSM was as follows:

$$\tilde{T}\approx \bar{T}+\phi b=\bar{T}+\sum_{k=1}^{t}\;b_{k}\phi_{k}$$

where $\phi =\left(\phi_{1},\phi_{2}\ldots \phi_{t}\right),b={\left(b_{1},b_{2}\ldots b_{t}\right)}^{⊤}$. By applying limits of $-c\sqrt{\lambda_{k}}\leq b_{k}\leq c\sqrt{\lambda_{k}}$ to the parameter $b_{k}$ and ∣c∣⩽3, we ensured that the shape generated was similar to that in the original data set.

#### 2.2.1 Morphological Analysis and Anatomical Measurements

**FIGURE 3:**
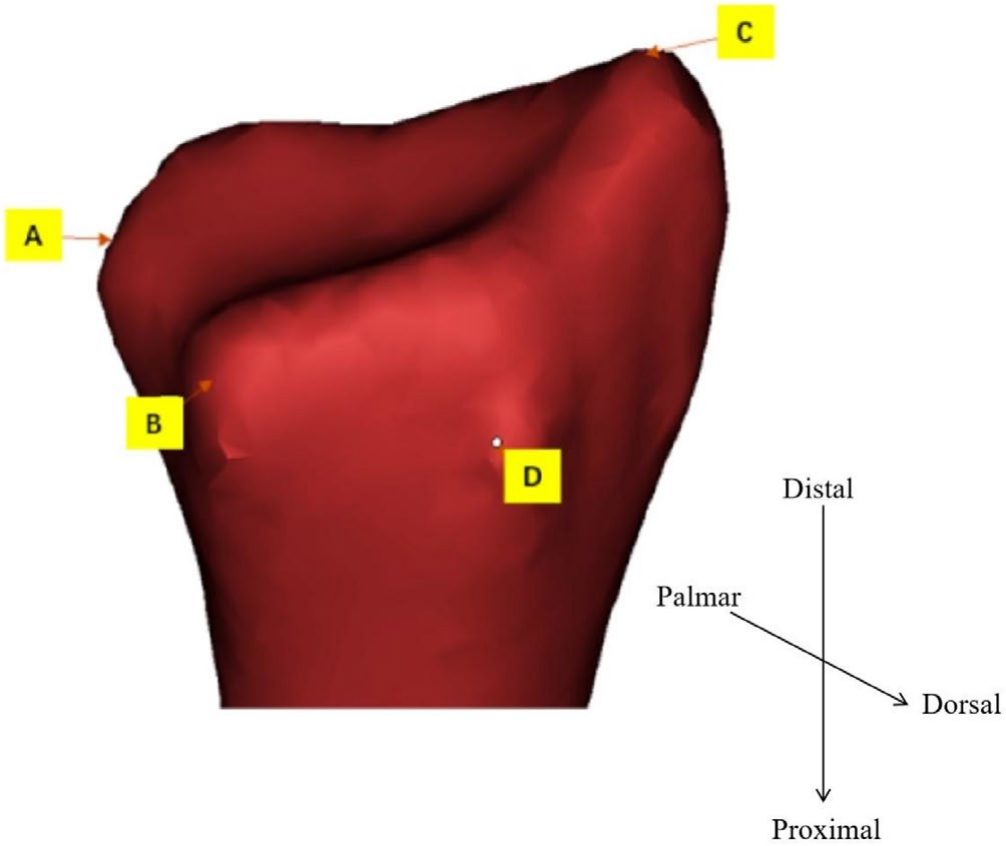
Location map of indicator markers (A, B, C, D). A: Sigmoid notch volar lips; B: Sigmoid notch dorsal lips; C: Radial styloid process; C: Styloid process vertex; D: The highest point on the dorsal side of Lister's tubercle.

Statistical shape models (SSMs) of the distal radius were constructed using principal component analysis (PCA) to evaluate the morphological variations among different groups (Figure 3). The analysis focused on characterizing the distinct features of normal and fractured specimens, including Barton's, Colles', and Smith's fractures.

To quantitatively assess the morphological differences, six anatomical indicators were carefully selected based on their clinical significance (Figure 2). These measurements included four distance parameters and two angular measurements. The distance parameters were measured between key anatomical landmarks: the volar (A) and dorsal (B) lips of the sigmoid notch (AB), the dorsal lip of the sigmoid notch to the styloid process vertex (bc), the volar lip of the sigmoid notch to the styloid process vertex (AC), and the styloid process vertex to the highest point of Lister's tubercle (CD). The angular measurements included the angle between the lunate facet radial crest line and the radius long axis (ALF), and the angle between the sigmoid notch midpoint‐styloid process line and the radius long axis (ASR).

All anatomical landmarks were identified by experienced orthopedic surgeons following standardized protocols to ensure measurement consistency and reliability. The measurements were performed using validated software tools that provided a precision of 0.1 mm for distance measurements and 0.1° for angular measurements. To ensure the reliability of the measurements, all landmarks were identified independently by two experienced surgeons, with any discrepancies resolved through consensus.

**FIGURE 4:**
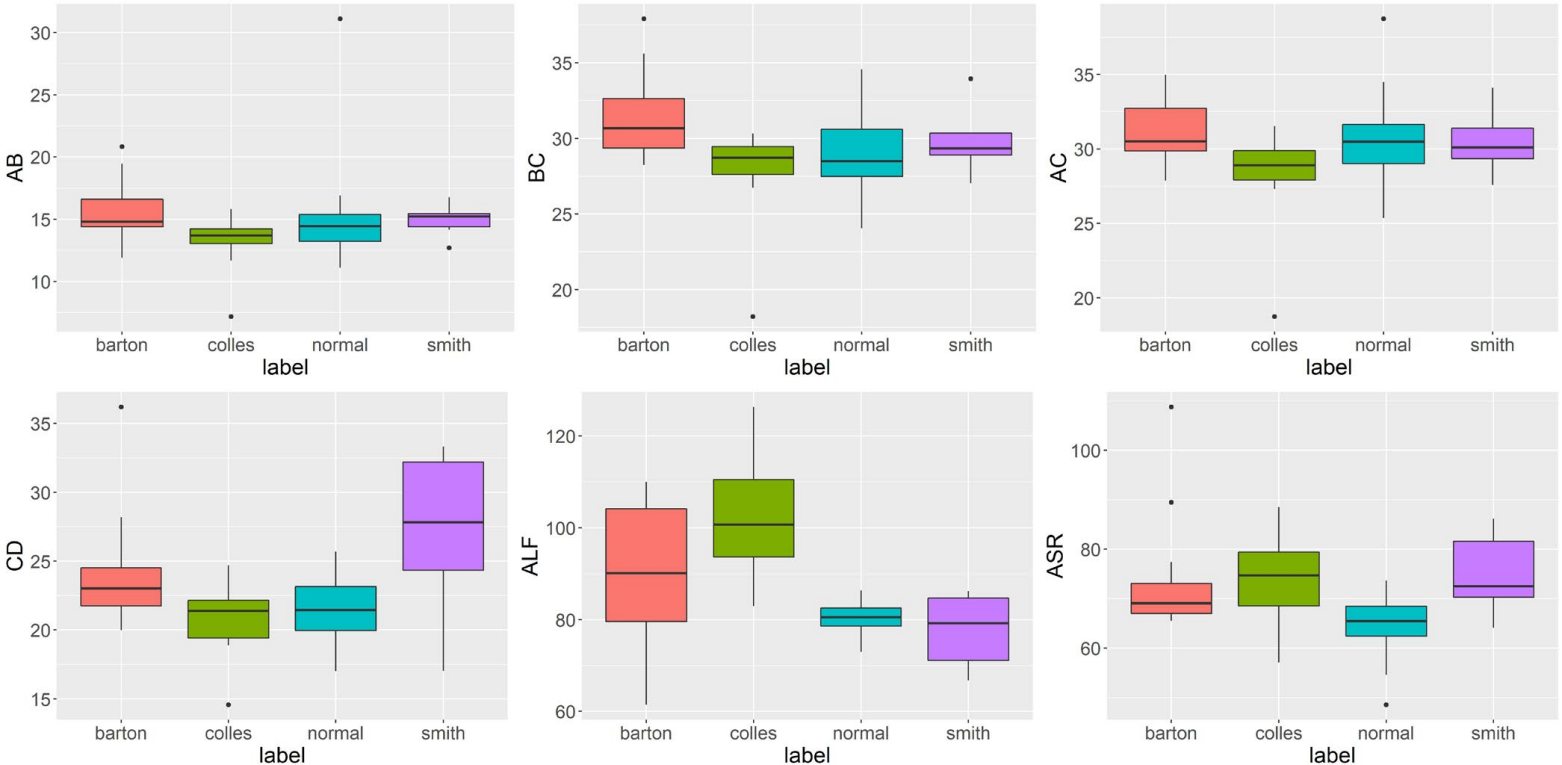
Boxplot of six indicators (AB, bc, AC, and CD [in mm] and ALF and ASR [in °]). (a) Normal; (b) Barton's fracture; (c) Colles' fracture; (d) Smith's fracture. AB: Straight‐line distance between the volar and dorsal lips of the sigmoid notch; bc: Straight‐line distance between the dorsal lips of the sigmoid notch and the vertex of the styloid process; AC: Straight‐line distance between the volar lips of the sigmoid notch and vertex of the styloid process; CD: Straight‐line distance between the styloid process vertex and the highest point on the dorsal side of the Lister's tubercle. ALF:Angle between the line connecting the highest point of the lunate facet radial crest at the palmar and dorsal sides and the long axis of the radius; ASR: The angle between the sigmoid notch midpoint and the line between the radial styloid process and the long axis of the radius.

**FIGURE 5:**
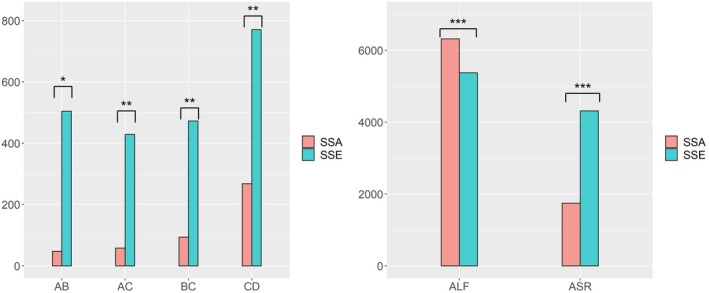
AB, bc, AC, CD [in mm]; ALF and ASR [in °] six indicator ANOVA histogram. Significance: 0 “***” 0.001 “**” 0.01 “*”. AB: Straight‐line distance between the volar and dorsal lips of the sigmoid notch; bc: Straight‐line distance between the dorsal lips of the sigmoid notch and the vertex of the styloid process; AC: Straight‐line distance between the volar lips of the sigmoid notch and vertex of the styloid process; CD: Straight‐line distance between the styloid process vertex and the highest point on the dorsal side of the Lister's tubercle. ALF:Angle between the line connecting the highest point of the lunate facet radial crest at the palmar and dorsal sides and the long axis of the radius; ASR: The angle between the sigmoid notch midpoint and the line between the radial styloid process and the long axis of the radius.

Statistical analysis was conducted using one‐way ANOVA followed by post hoc Tukey tests to compare the measurements across the four groups (Figures 4 and 5). Statistical significance was established at three levels: *p* < 0.05, *p* < 0.01, and *p* < 0.001, allowing for a detailed comparison of morphological differences between groups.

### 2.3 Neural Network Classifier

NNs can be devoted to pattern classification [33]. The basic unit of NN has three layers, including the input, hidden, and output layers. The number of neurons in the input layer was the characteristic number in the SSM; the hidden layer neurons were obtained by combining linear‐weighting input layer neurons with the sigmoid activation function, and the output layer neurons were similar. In the learning stage, the hidden layer neurons were represented as follows:

$$H=\text{sigmoid}\left(W^{T}\phi +B\right)$$

The output layer neurons were represented as follows:

$$P=\text{sigmoid}\left(V^{T}H+C\right)$$

Cross entropy loss function optimization is given as follows:

$$\text{Loss}\left(L,P\right)=-\sum_{i}^{n}L_{i}\log\left(P_{i}\right)$$

where L was the true sample label, P was the predicted label. The stochastic gradient descent algorithm was used to learn the parameters $\theta =\left(W,V,B,C\right)$ of the NN model.

### 2.4 Statistical Analysis

Prior to conducting ANOVA, we verified the required statistical assumptions. The Shapiro–Wilk test was performed to assess the normality of the data distribution for both normal and fracture groups. Results showed no significant deviation from normality for the primary morphological parameters (normal group: *W* = 0.976, *p* = 0.512; fracture group: *W* = 0.969, *p* = 0.384). Homogeneity of variances was confirmed using Levene's test (*F* = 1.842, *p* = 0.178), indicating that the variance between groups was not significantly different.

The ANOVA analysis revealed significant differences between normal and fracture groups in several key morphological parameters. Post hoc power analysis indicated sufficient statistical power (1 − *β* = 0.92) for detecting medium to large effect sizes (*η* ^2^ = 0.15) at α = 0.05. For parameters showing significant differences (*p* < 0.05), we conducted Tukey's HSD test for multiple comparisons to identify specific group differences while controlling for the family‐wise error rate.

## 3 Results

### 3.1 Study Population Characteristics

Before presenting the analytical results, we first describe the baseline characteristics of our cohort. The study population comprised 80 patients (43 controls / 37 fractures) with a mean age of 56.3 ± 8.7 years, of whom 71.3% (*n* = 57) were female. Key demographic and clinical parameters showed balanced distributions between groups, as shown in Table 1.

**TABLE 1:** Baseline characteristics of study population.

Parameter	Control (*n* = 43)	Fracture (*n* = 37)	*p*
Age (years)	55.8 ± 8.4	56.9 ± 9.1	0.58
Female, *n* (%)	30 (69.8%)	27 (73.0%)	0.75
BMI (kg/m^2^)	24.3 ± 3.2	24.8 ± 3.5	0.51
Dominant hand involved, n(%)	24 (55.8%)	20 (54.1%)	0.87

The participants were divided into two groups: control group (*n* = 43) and fracture group (*n* = 37). Continuous variables were compared using Student's t‐test, categorical variables with *χ* ^2^ test (SPSS v26.0). Key demographic and clinical parameters showed balanced distributions between groups. The control group had a mean age of 55.8 ± 8.4 years, with 69.8% (*n* = 30) female participants and 55.8% (*n* = 24) dominant hand involvement. The fracture group showed similar characteristics, with a mean age of 56.9 ± 9.1 years (*p* = 0.58), 73.0% (*n* = 27) female participants (*p* = 0.75), and 54.1% (*n* = 20) dominant hand involvement (*p* = 0.87). BMI was also comparable between groups (24.3 ± 3.2 vs. 24.8 ± 3.5, *p* = 0.51).

In the fracture group, CT scans were obtained within 24 h of injury in 25 cases, between 24 and 72 h in 9 cases, and after 72 h in three cases. Morphological fracture classification revealed: Colles' fracture accounted for 62% (*n* = 23), Smith's fracture for 24% (*n* = 9), and Barton's fracture for 14% (*n* = 5).

### 3.2 Classification Model Performance and Cross‐Validation

**FIGURE 6:**
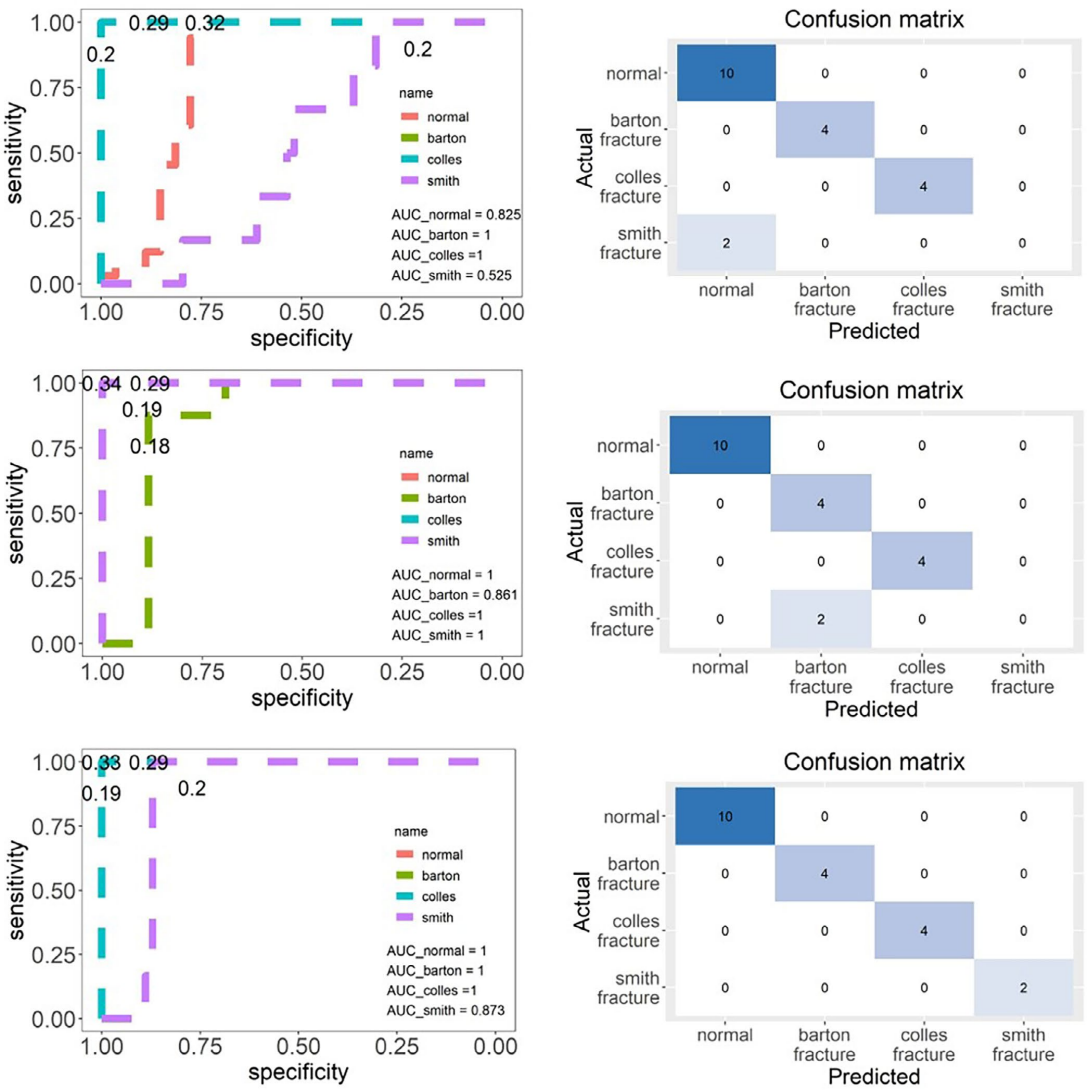
AUC of the training dataset and confusion matrix of the test dataset in 2th‐fold cross‐validation when the number of main features was 5, 10, and 15. AUC: The area under receiver operational characteristic curve.

**FIGURE 7:**
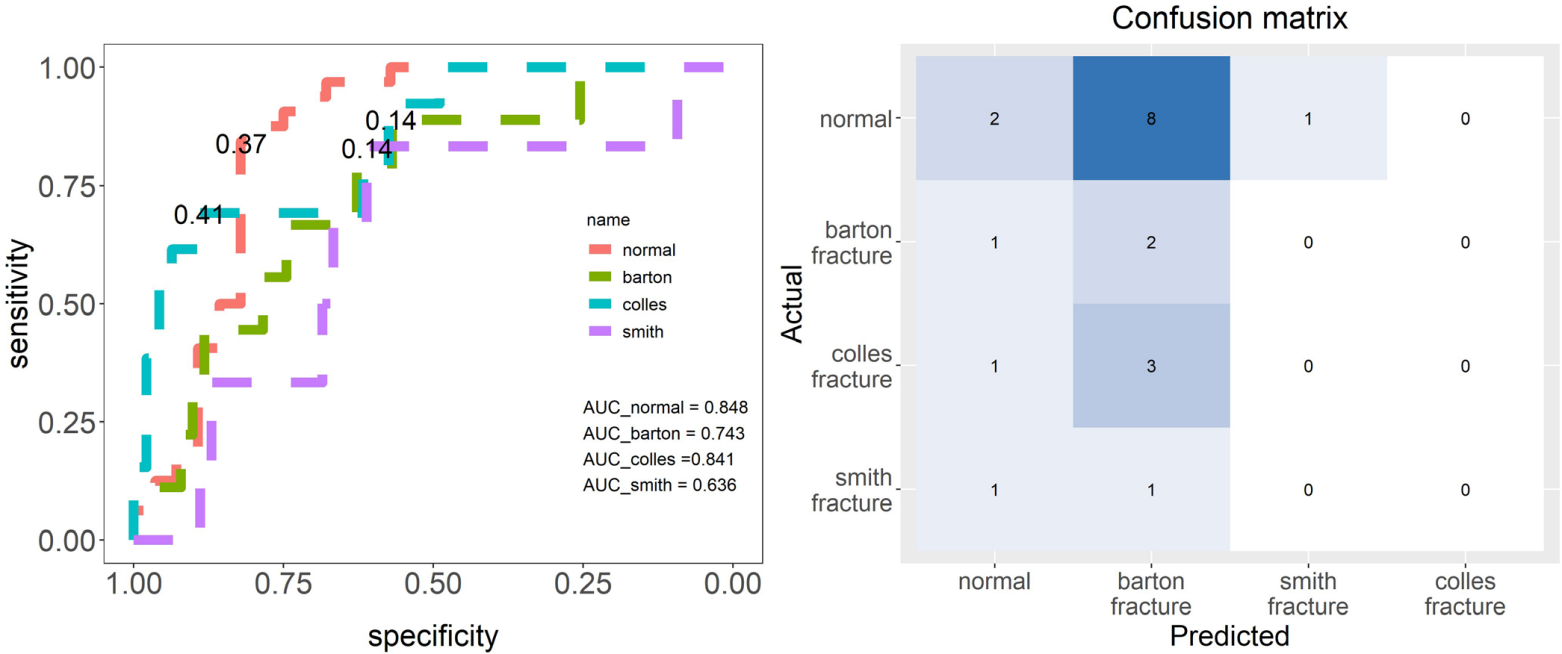
AUC of the training dataset and confusion matrix of the test dataset with the six indicator features. AUC: The area under receiver operational characteristic curve.

We selected a classifier to discriminate intelligently based on SSM features and analyzed the influence of different feature numbers on the discrimination results. Table 2 reveals that when the number of characteristics is 15, the cumulative contribution rate of variance is > 75%. This reveals that extracting the first 15 features can explain all the data. A four‐fold cross‐validation method was used to verify the robustness of the classifier and prevent the influence of data selection on the results. Table 3 reveals that the area under the curve (AUC) of each training dataset was > 95% based on the > 10 features in the one‐ and four‐fold training data. However, when the eigenvalue was > 10, AUC values were higher (Table 3 and Figure 6). The optimal prediction threshold was obtained from the test set and receiver operating characteristic (ROC) curve in (Table 4 and Figure 6). In the discriminant analysis of whether it was a normal group, if $P_{i}>0.333$, then the individual was deemed normal. In the discriminant analysis of whether it was a Barton's fracture, if $P_{i}>0.197$, then the individual was concluded to have a Barton's fracture. In the discriminant analysis of whether it was a Colles' fracture, if $P_{i}>0.286$, then the individual was judged as having a Colles' fracture. In the discriminant analysis of whether it was a Smith's fracture, if $P_{i}>0.197$, then individual was judged as having a Smith's fracture. The discrimination confusion matrix of each group is illustrated in Figure 6. Table 5 demonstrates that increasing the number of features from 5 to 15 improved the classification accuracy, with the highest mean accuracy of 0.975 achieved with 15 features. The selection of more features correlated with a higher accuracy of the test set discrimination. Figures 6 and 7 reveal that > 10 features extracted using PCA had a better classification effect than the defined six indicators.

**TABLE 2:** Variance cumulative contribution rate with different groups when the number of main features is 5, 10, and 15, respectively.

Feature	*F* = 5	*F* = 10	*F* = 15
Normal	53.24%	65.93%	75.11%
Barton fracture	70.58%	96.16%	100.00%
Colles fracture	65.68%	87.03%	97.80%
Smith fracture	91.85%	100.00%	100.00%

**TABLE 3:** The value of AUC with train dataset different group by 4‐fold cross‐ validation when the number of main features is 5, 10 and 15 respectively.

	AUC	Normal	Barton fracture	Colles fracture	Smith fracture	Mean
	k_1	0.838	1	1	0.512	0.838
*F* = 5	k_2	0.825	1	1	0.525	0.838
	k_3	0.823	1	1	0.574	0.849
	k_4	0.817	1	1	0.494	0.828
	k_1	1	0.882	1	1	0.971
*F* = 10	k_2	1	0.861	1	1	0.965
	k_3	1	0.882	1	1	0.971
	k_4	1	0.880	1	1	0.970
	k_1	1	1	1	0.864	0.966
*F* = 15	k_2	1	1	1	0.873	0.968
	k_3	1	1	1	0.864	0.966
	k_4	1	1	1	0.836	0.959

**TABLE 4:** The value of prediction threshold when the number of main features is 15.

Prediction threshold	*F* = 15	Normal	Barton fracture	Colles fracture	Smith fracture
	k_1	0.333	0.197	0.287	0.196
	k_2	0.334	0.194	0.288	0.197
	k_3	0.333	0.196	0.287	0.197
	k_4	0.332	0.200	0.282	0.199
	Mean	0.333	0.197	0.286	0.197

**TABLE 5:** The value of accuracy with test dataset different group by 4‐fold cross‐ validation when the number of main features is 5, 10 and 15 respectively.

ACC	k_1	k_2	k_3	k_4	Mean
*F* = 5	0.85	0.9	0.8	0.7	0.813
*F* = 10	0.85	0.9	0.9	0.8	0.863
*F* = 15	1	1	1	0.9	0.975

### 3.3 Statistical Analysis of Group Differences

The ANOVA results showed significant differences between groups for radial inclination (*F*(1,78) = 15.32, *p* < 0.001, *η* ^2^ = 0.164), volar tilt (*F*(1,78) = 18.76, *p* < 0.001, *η* ^2^ = 0.194), and radial height (*F*(1,78) = 12.45, *p* < 0.001, *η* ^2^ = 0.138). Post hoc comparisons using Tukey's HSD test revealed that the fracture group showed significantly reduced radial inclination (mean difference = 5.8°, 95% CI [3.9, 7.7]), volar tilt (mean difference = 6.4°, 95% CI [4.2, 8.6]), and radial height (mean difference = 3.2 mm, 95% CI [2.1, 4.3]) compared to the normal group.

## 4 Discussion

### 4.1 Novel SSM‐NN Approach for Fracture Classification

Our study demonstrated three key findings: (1) The SSM‐based intelligent classifier achieved a 97.5% accuracy rate in distinguishing between normal distal radius and three types of fractures (Colles', Smith's, and Barton's); (2) The optimal classification performance was achieved using the first 15 PCA‐extracted features, with high robustness confirmed through four‐fold cross‐validation; (3) The CT‐based SSM approach provided comprehensive three‐dimensional morphological analysis, offering advantages over traditional X‐ray‐based methods for fracture classification. In this study, we successfully established an SSM‐based intelligent classifier for normal and DRFs (Colles', Smith's, and Barton's fractures) using CT data. The classifier achieved a 97.5% accurate diagnosis rate, with the first 15 PCA‐extracted features showing optimal classification performance. The four‐fold cross‐validation demonstrated the robustness of our approach, with high AUC values across all test sets [31]. The high accuracy and efficiency of our approach demonstrate significant clinical potential. While traditional fracture diagnosis relies heavily on clinicians' experience, our SSM + NN method provides objective, quantifiable morphological analysis. This standardized approach could be particularly valuable in teaching hospitals, helping young surgeons develop diagnostic skills and ensuring consistent fracture classification across different observers.

### 4.2 Advantages Over Existing Methods

Compared to previous studies using X‐ray‐based CNN models [16, 17, 32], our CT‐based SSM approach offers several advantages. The CT data provide more comprehensive morphological information, particularly for detecting subtle articular surface steps and rotational deformities [23, 24]. While Gan et al. [32] trained a DRFs diagnostic CNN model with 2340 anterior–posterior wrist X‐rays, and Raisuddin et al. [17] developed the DeepWrist program for identifying the presence or absence of DRF, our method successfully classifies different fracture types. The superior performance of our method with a smaller dataset has practical implications for clinical implementation. It makes the approach more feasible for adoption in various clinical settings, including smaller hospitals with limited data resources. Additionally, the CT‐based SSM analysis provides more detailed information about fracture morphology, which can directly inform surgical planning and treatment strategy selection (Table 6).

**TABLE 6:** Comparison between major intelligent diagnostic models for distal radius fractures.

Principal investigator	Types of classifiers	Function	Research materials	Data frame	AUC
Oka et al. {Oka, 2021 #33}	CNN	Presence of distal radius fracture	369 x‐ray AP images and 360 LAT images of distal radius fractures	VGG16	98.0% ± 1.6%
Suzuki et al. {Suzuki, 2022 #31}	CNN	Presence of distal radius fracture	503 cases of DRF diagnosed by radiographs and 289 cases without fractures	Keras and Tensorflow	99.3%
Gan et al. {Gan, 2019 #10}	CNN	Presence of distal radius fracture	AP wrist radiographs of 2340 patients	Faster R‐CNN and Inception‐v4	96%
Raisuddin et al. {Raisuddin, 2021 #59}	CNN	Presence of distal radius fracture	3873 AP and LAT wrist X‐ray images, including 953 cases of distal radius fractures	GradCAM and KNEEL	99%
Kim et al. {Kim, 2018 #60}	CNN	Presence of wrist fracture	After an eight‐fold data enhancement technique, a total of 11,112 lateral wrist radiographs were trained from the initial 1389 radiographs (695 “fractures” and 694 “no fractures”)	Inception v3 network	95.4%, setting the diagnostic cutoff at thresholds designed to maximize sensitivity and specificity, resulted in 90% and 88%, respectively.
Kim et al. {Kim, 2021 #61}.	CNN	Presence of wrist fracture	4551 radiographs from 798 patients and 4443 wrist radiographs from 1481 patients with and without fractures	DenseNet‐161 and ResNet‐152	DenseNet‐161:96.2%
					ResNet‐152: 94.7%

### 4.3 Morphological Analysis and Clinical Implications

Our findings extend the work of Baumbach et al. [31], who established SSM for normal distal radius morphology. The main morphological differences we identified between normal and fractured distal radius included variations in the straight‐line distances between key anatomical landmarks and specific angular measurements [25, 26, 28, 34]. These findings provide valuable insights into the geometric characteristics of different fracture patterns.

### 4.4 Future Research Directions

Building upon our current findings, several promising future research directions warrant investigation. First, integrating soft tissue information and bone density data could provide a more comprehensive understanding of fracture mechanisms. This could be achieved through a multi‐modal imaging approach combining CT, MRI, and DXA scans, potentially revealing the complex interplay between bone geometry, mineral density, and surrounding tissue properties in fracture development.

Transfer learning presents another exciting avenue for research. With the emerging availability of large‐scale medical imaging datasets, pre‐trained models could be fine‐tuned on distal radius data, potentially improving model performance while reducing the required sample size for effective training. This approach could be particularly valuable for medical centers with limited data resources.

Multi‐center validation studies represent a crucial next step. By collecting data from diverse geographical locations and patient populations, we can evaluate the model's generalizability and identify potential population‐specific variations in fracture patterns. This could lead to more robust and widely applicable prediction models.

Integration with clinical decision support systems offers practical implementation possibilities. Future work could focus on developing user‐friendly interfaces that combine our morphological analysis with other clinical parameters, providing real‐time risk assessment tools for clinicians. This integration could include automated reporting systems and personalized prevention strategy recommendations based on individual patient morphology.

### 4.5 Limitations and Strengths

While our sample size (43 normal, 37 fractures) is sufficient for SSM‐based analysis as demonstrated by the high classification accuracy (97.5%) and robust statistical power (0.83), future studies with larger cohorts would be valuable for further validation across diverse populations and fracture patterns. The current sample size, though adequate for establishing model feasibility and initial clinical validation, could be expanded to enhance generalizability. Although our method demonstrated high accuracy with this limited dataset, the small sample size could potentially impact the robustness of our findings when applied to more diverse patient populations. To minimize potential biases, we implemented several measures, including balanced case stratification and rigorous four‐fold cross‐validation, which showed consistent performance across different data subsets. Compared to similar studies in the field, our sample size is within the typical range, though we acknowledge that a larger dataset would strengthen our conclusions. Second, while we identified that 15 PCA‐extracted features provided optimal classification, the specific nature of these features requires further investigation. Third, the current dataset comes from a single medical center, which may introduce selection bias, particularly in terms of patient demographics and fracture patterns. Additionally, our current SSM model only considers geometric morphological features without incorporating other important clinical parameters such as bone density. Future multi‐center studies with larger, more diverse samples and integrated clinical parameters will be valuable for validating and extending our findings. However, the high accuracy achieved with this sample size demonstrates the method's efficiency in feature extraction and pattern recognition. The SSM + NN approach's ability to perform well with limited data suggests its potential robustness in clinical applications, though further validation with diverse cases is needed.

Despite these limitations, our study has several notable strengths. First, we developed an innovative approach combining SSM with NN for DRF classification. This method analyzes comprehensive three‐dimensional morphological features rather than solely relying on visible displacement, making it theoretically more sensitive to subtle geometric changes—particularly valuable for early‐stage fractures or cases with minimal displacement where visual diagnosis might be challenging [17, 32]. Second, unlike traditional CNN‐based methods requiring massive training datasets, our SSM + NN approach achieves high accuracy (97.5%) with a relatively small sample size, demonstrating superior efficiency in both computational power and data requirements [9, 10, 11, 12, 13, 14, 15]. The dimensional reduction nature of SSM (from 2000 points to 15 principal components) enables effective feature extraction and classification with fewer samples while maintaining robust performance.

## 5 Conclusions

In conclusion, based on CT data, an SSM with normal and fracture conditions of the distal radius was established for the first time. The SSM combined with NN not only provides an intelligent diagnostic classifier with high accuracy but also offers a standardized, objective approach to fracture analysis. This method shows promise for clinical application, particularly in supporting diagnostic decision‐making and surgical planning. While further validation is needed, our findings establish a solid foundation for the intelligent diagnosis of clinical fractures and suggest potential applications in both clinical practice and surgical training.

## Author Contributions

The authors of this study contributed to the research in various aspects. Xing‐bo Cai and Ze‐hui Lu participated in the main experimental design, data collection, and manuscript writing. Zhi Peng and Yong‐qing Xu contributed to data preprocessing and model establishment. Jun‐shen Huang, Hao‐tian Luo, and Yu Zhao contributed to model establishment and experimental results analysis. Zhong‐qi Lou, Zi‐qi Shen, Zhang‐cong Chen, and Xiong‐gang Yang contributed to data preprocessing and experimental results analysis. Ying Wu and Sheng Lu were the administrators of theproject.

## Ethics Statement

The study was conducted in accordance with the Declaration of Helsinki and approved by the Institutional Review Board of the First People's Hospital of Yunnan Province. Informed consent was obtained from all subjects involved in the study.

## Consent

All authors read and approved the final manuscript.

## Conflicts of Interest

The authors declare no conflicts of interest.

## Supporting information


**Data S1.** SSM construction example.

## Data Availability

The datasets used and/or analyzed during the current study are available from the corresponding author upon reasonable request.
