# Supplementary material for: A Neural Network Model for Intelligent Classification of Distal Radius Fractures Using Statistical Shape Model Extraction Features
Source: Orthop Surg. 2025 Apr 3;17(5):1513–24. doi: 10.1111/os.70034 (PMC12050184; doi:10.1111/os.70034)
Supplement: Supplementary file 1 — Data S1. SSM construction example. [file OS-17-1513-s001.docx]

**Supplementary Material 1:** SSM Construction Example

Using participant #001 (female, 65 years old) as an example, we demonstrate the complete SSM construction process:

1. Initial Point Cloud Data:

Raw CT scan parameters:

- Slice thickness: 0.625 mm

- Matrix size: 512 × 512

- Pixel spacing: 0.351 mm

Initial point cloud coordinates (partial list, total 2000 points):

P1: (125.3, -45.2, 78.9)

P2: (124.8, -44.9, 78.7)

P3: (125.1, -45.5, 78.4)

...

P2000: (123.9, -46.1, 77.8)

2. After Registration:

Registration parameters:

- Initial translation vector: [2.5, -1.2, 0.8] mm

- Rotation matrix: [0.998, -0.052, 0.031; 0.052, 0.998, -0.015; -0.031, 0.016, 0.999]

- Final RMS error: 0.42 mm

Registered coordinates (partial list):

P1': (127.8, -46.4, 79.7)

P2': (127.3, -46.1, 79.5)

P3': (127.6, -46.7, 79.2)

...

P2000': (126.4, -47.3, 78.6)

3. PCA Calculation Process:

Mean shape vector μ (partial):

μ = [127.5, -46.3, 79.4, ..., 126.5, -47.2, 78.7]

First three eigenvalues:

λ1 = 245.6

λ2 = 156.3

λ3 = 98.7

Cumulative variance explained:

- First component: 42.3%

- First two components: 69.1%

- First three components: 86.2%

4. Final SSM Parameters:

Shape parameters for participant #001:

b1 = 1.24

b2 = -0.56

b3 = 0.31

...

b10 = 0.02

Model limits (for first mode):

-2.7√λ1 ≤ b1 ≤ 2.7√λ1

Reconstruction accuracy:

- Mean surface distance: 0.38 mm

- Maximum surface distance: 1.12 mm

- Standard deviation: 0.21 mm
